# Long-term deer exclosure alters soil properties, plant traits, understory plant community and insect herbivory, but not the functional relationships among them

**DOI:** 10.1007/s00442-017-3895-3

**Published:** 2017-07-01

**Authors:** Jörg G. Stephan, Fereshteh Pourazari, Kristina Tattersdill, Takuya Kobayashi, Keita Nishizawa, Jonathan R. De Long

**Affiliations:** 1 Department of Ecology, Swedish University of Agricultural Sciences, 750 07 Uppsala, Sweden; 2 Department of Crop Production Ecology, Swedish University of Agricultural Sciences, 750 07 Uppsala, Sweden; 3 Department of Aquatic Sciences and Assessment, Swedish University of Agricultural Sciences, 750 07 Uppsala, Sweden; 4 Department of Zoology, Graduate School of Science, Kyoto University, Kitashirakawa-oiwake, Sakyo, Kyoto, 606-8502 Japan; 5 Department of Environment and Natural Sciences, Graduate School of Environment and Information Sciences, Yokohama National University, 79-7 Tokiwadai, Hodogaya, Yokohama, Kanagawa 240-8501 Japan; 6 School of Earth and Environmental Sciences, The University of Manchester, Manchester, M13 9PT England UK; 7 Department of Forest Ecology and Management, Swedish University of Agricultural Sciences, 901 83 Umeå, Sweden

**Keywords:** Trophic cascade, Phenotypic plasticity, Herbivore load, Plant diversity, Plant defense

## Abstract

Evidence of the indirect effects of increasing global deer populations on other trophic levels is increasing. However, it remains unknown if excluding deer alters ecosystem functional relationships. We investigated how sika deer exclosure after 18 years changed soil conditions, the understory plant community, the traits of a dominant understory plant (*Sasa palmata*), herbivory by three insect-feeding guilds, and the functional relationships between these properties. Deer absence decreased understory plant diversity, but increased soil organic matter and ammonium concentrations. When deer were absent, *S. palmata* plants grew taller, with more, larger, and tougher leaves with higher polyphenol concentrations. Deer absence led to higher leaf area consumed by all insect guilds, but lower insect herbivory per plant due to increased resource abundance (i.e., a dilution effect). This indicates that deer presence strengthened insect herbivory per plant, while in deer absence plants compensated losses with growth. Because plant defenses increased in the absence of deer, higher insect abundances in deer absence may have outweighed lower consumption rates. A path model revealed that the functional relationships between the measured properties were similar between deer absence versus presence. Taken together, deer altered the abiotic and biotic environment, thereby changing insect herbivory, which might impact upon nutrient cycling and primary productivity. These results provide evidence that deer can alter interactions between trophic levels, but that functional relationships between certain ecosystem components may remain constant. These findings highlight the need to consider how increasing global deer populations can have cascade effects that might alter ecosystem dynamics.

**Electronic supplementary material:**

The online version of this article (doi:10.1007/s00442-017-3895-3) contains supplementary material, which is available to authorized users.

## Introduction

Dramatic increases in deer populations have become a global issue (Côté et al. 2004; Takatsuki 2009a; Warren 2011). The presence of deer can impact on ecosystem processes such as successional trajectories (Gill and Beardall 2001), nitrogen (N) and phosphorus budgets (Abbas et al. 2012) and landscape-level water cycling (Hobbs 1996). Further, deer grazing alters plant community composition (Abrams and Johnson 2012; Habeck and Schultz 2015; Perea et al. 2014), as deer selectively graze on preferred species, which allows unpalatable species to proliferate (Takatsuki and Itô 2009; Wardle et al. 2001). Additionally, the effect of deer grazing on tree seedling establishment can be positive (i.e., reduce competition with other vegetation) or negative (i.e., deer selectively browse seedlings) (Itô and Hino 2005). Therefore, studying the effects of deer herbivory is crucial to understanding how ecosystem functioning could be altered as climate and land use change continue to impact on deer populations globally.

Damage done directly to plants by large herbivores such as deer often induces fundamental phenotypic changes to both the physical and chemical traits of a plant (Danell and Huss-Danell 1985; Karban 2011; Ohgushi 2005). Deer browsing can reduce plant traits, such as plant height, number of shoots (Den Herder et al. 2004), number of leaves per plant (Takagi and Miyashita 2012), and leaf N content (Lind et al. 2012). Nonetheless, occasionally deer browsing may actually increase foliar nutrient content (Takagi and Miyashita 2012). Further, deer herbivory can reduce chemical defense traits such as foliar tannin concentrations (Barrett and Stiling 2007; Shimazaki and Miyashita 2002), while sometimes deer herbivory can increase plant structural defense (Shikata et al. 2013). In some cases, deer herbivory results in compensatory growth (Takagi and Miyashita 2012) and can even increase reproductive output (Ohgushi 2005).

In addition to these direct effects, deer herbivory can induce changes to both soil and plant properties that might indirectly affect interactions with other trophic groups. For example, Kardol et al. (2014) found that deer impacts on the structure of the soil affected the mycorrhizal community, thereby generating negative effects on tree seedling establishment. Takatsuki and Itô (2009) showed that high densities of deer inhibited tree regeneration and favored understory plant communities composed of highly herbivory-tolerant and well-defended species. Furthermore, changes to plant properties induced by deer presence can have positive (Barrett and Stiling 2007; Takagi and Miyashita 2012) or negative (Lind et al. 2012; Shimazaki and Miyashita 2002) effects on the attack of these plants by other herbivores. The direction of these indirect plant-mediated interactions induced by deer can be similar or different across different insect-feeding guilds (Poelman et al. 2010; Viswanathan et al. 2005). The response of plant traits to deer herbivory and the impact that such responses might have on the likelihood of future insect herbivore attack has been explored (see references above). However, there is a dearth of knowledge concerning how deer herbivory might initiate multi-trophic cascades (Martin et al. 2011; Nuttle et al. 2011), with subsequent effects on ecosystem function and processes.

Recently, the effect of deer grazing on multiple trophic groups has been gaining greater attention (Cardinal et al. 2012; Côté et al. 2004; Davalos et al. 2015; Foster et al. 2014). In Japan, the Sika deer (*Cervus nippon*), which is found on all the major islands, has expanded its range by 70% in recent decades (Nakajima 2007). Increased sika deer grazing favors the dominance of the unpalatable shrub *Berberis thunbergii*, which in turn benefited the Japanese Macaque (*Macaca fuscata*) that feeds upon its berries (Tsuji and Takatsuki 2004). However, in another study sika deer grazing reduced the arthropod community as a result of decreased understory plant cover (Katagiri and Hijii 2015). Furthermore, deer grazing may influence the abundance and diversity of insect herbivores by affecting the growth and development of their host (Suominen et al. 1999). Oviposition and larval weight of the gall midge *Procystiphora uedai* that typically uses *Sasa* species as a host was negatively impacted on by sika deer browsing (Tabuchi et al. 2010). Despite increasing knowledge on how deer grazing impacts upon other organisms across different trophic levels, there is still a lack of knowledge concerning how deer affect insect herbivory across different feeding guilds in the understory vegetation.

In deciduous broad-leaved forests in the temperate regions of Japan, the understory vegetation is often dominated by *Sasa* species, also known as dwarf bamboo (Miyawaki et al. 1982). The *Sasa* species are perennial, semi-woody and rhizomatous plants that typically reproduce vegetatively, with rare mast flowering events occurring every few decades (Abe and Shibata 2012; Makita 1992). After these mast flowering events, nutrients sequestered in *Sasa* species biomass are released into the soil, subsequently providing an important source of nutrients for trees and tree seedlings (Tripathi et al. 2005). A number of insects depend upon *Sasa* species as host plants, including Lepidoptera species (Ide 2004), gall midges (Tabuchi et al. 2010) and leaf hoppers (Matsukura et al. 2009). Sika deer also commonly consume *Sasa* species, especially during the winter when other annual understory vegetation is unavailable (Takatsuki 2009b).

In this study, we aimed to gain understanding of multi-trophic interactions initiated by deer. We measured soil properties, the understory plant community, and the traits of the dominant understory plant, *Sasa palmata*, both inside and outside a deer exclosure fence that had been in place for 18 years. Our first objective was to determine how deer herbivory might lead to alterations in feeding patterns in three insect-feeding guilds. To our knowledge, no study to date has sought to disentangle the direct and indirect causal effects of deer presence on insect herbivores (Kardol et al. 2014). Our second objective was to gain an understanding of the general relations among the abiotic and biotic (including insect herbivory) properties of the ecosystem and to see if these relationships were altered due to deer exclosure.

**Fig. 1:**
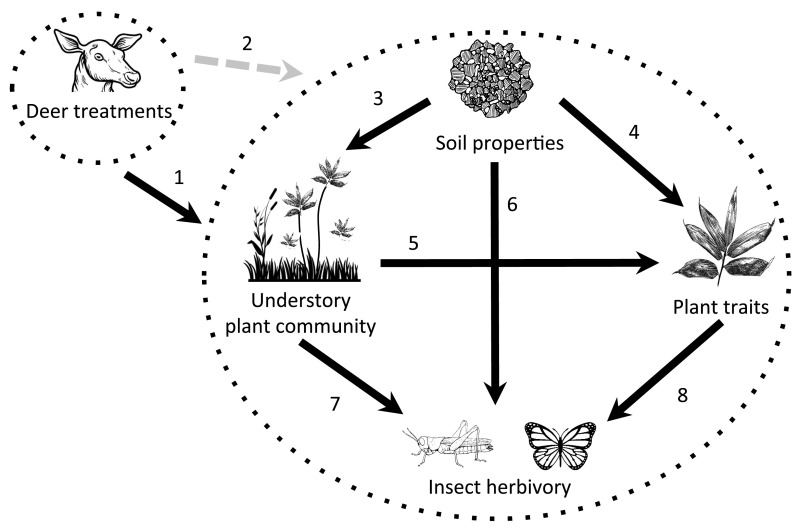
A priori model showing the potential effects of deer on ecosystem properties and functional relationships. *Arrow numbers* indicate different pathways between ecosystem properties and are referenced in *bold* below. Deer can impact on a number of ecosystem components including insect herbivory (Yamazaki and Sugiura 2008), soil properties (Abbas et al. 2012), plant traits (Karban 2011; Ohgushi 2005) and the understory plant community (Takatsuki and Itô 2009; Wardle et al. 2001) (*1*). Further, deer could alter the functional relationships between these components (which we test here) (*2*). Soil properties that have been changed by deer can affect the understory plant community (*3*, Kardol et al. 2014) and plant traits (*4*, Pullin and Gilbert 1989), while the understory plant community can also impact on plant traits (*5*, Takagi and Miyashita 2015). Soil properties (*6*, Wardle et al. 2001), the understory plant community (*7*, Katagiri and Hijii 2015) and plant traits (*8*, Lind et al. 2012; Ohgushi 2005; Tabuchi et al. 2010) could all act as direct or indirect mediators of insect herbivory via changes caused by deer

We hypothesized that: (1) insect herbivory on *S. palmata* will increase in the absence of sika deer grazing. This is because the absence of deer herbivory typically leads to increased foliar nutrient concentrations (Lind et al. 2012), lower leaf toughness (Coley 1983; Lambers and Poorter 1992) and decreased plant defense (Shikata et al. 2013), which is favorable to insect herbivores (Lind et al. 2012; Takagi and Miyashita 2015); (2) more *S. palmata* individuals per area as the result of deer absence (Nishizawa et al. 2016) will dilute the increase in insect herbivory, leading to lower insect herbivory per plant; (3) damage by different insect-feeding guilds to *S. palmata* will be affected differently by sika deer grazing due to disparate resource requirements between guilds (i.e., specialist versus generalist herbivores) (Barrett and Stiling 2007); (4) the functional relationships between soil properties, the plant community and the traits of *S. palmata* will be altered due to deer exclosure, thereby impacting on insect herbivory. This is because deer presence is known to alter plant traits (Karban 2011; Ohgushi 2005), soil properties (Abbas et al. 2012; Kardol et al. 2014) and the understory plant community (Nishizawa et al. 2016; Takatsuki and Itô 2009; Wardle et al. 2001) in ways that can drive insect herbivory via indirect trophic interactions (Fig. 1). We expected deer to have a disproportionate influence on different components of the ecosystem. For example, deer absence could lower plant-available soil N leading to higher plant defense and resulting in lower insect herbivory, but the impact on insect herbivory may not be in proportion to the decrease in N availability. Investigating how sika deer impact upon insect herbivory in *Sasa* species and changes the functional relationships between ecosystem components will further our understanding of how deer herbivory alters different trophic groups and ecosystem functioning.

## Methods

### Study site

The study was conducted in Nikko National Park, Japan (36°46′26.59″N, 139°25′13.94″E) in a deciduous cool-temperate forest. The dominant overstory species is *Quercus crispula* and the understory is dominated by *S. palmata* (Uchida et al. 2008). Between 2004 and 2014, mean annual air temperature was 7.1 °C and mean annual precipitation was 2277 mm (http://www.data.jma.go.jp/obd/stats/etrn/index.php; Uchida et al. 2008). The dominant soil type is dark brown forest soil (Forestry Agency 1983).

In 1997 a deer-proof fence (mesh size: 15 × 15 cm) was constructed around a section of the national park to protect the vegetation from deer grazing (Okuda et al. 2014). Sika deer density outside the fence has fluctuated between 13 and 22 animals per km^−2^ between 1996 and 2011 (Seto et al. 2015) and deer were completely excluded from the inside of the fence at our site for 18 years before the study commenced, while other smaller herbivorous animals, such as rodents, were likely able to pass through the fence. Sika deer presence outside of the fence was confirmed using camera traps.

### Experimental setup

A total of 24 plots measuring 1 × 1 m were established on 30-Sep-2015 and 1-Oct-2015. The plots were on both deer-present and deer-absent sides of the fence (12 on each side), with an average distance of 10 m between plots on each side. All plots in both the deer-present and deer-absent treatments were located approximately 20 m from the fence. The selected plots were representative of the understory plant community of the study site and contained no large trees. These plot selection criteria are typical for such large herbivore exclosure experiments and are considered appropriate to help control for confounding factors such as topography, understory plant community composition and aspect, while ensuring adequate independence between plots (Schrama et al. 2013; te Beest et al. 2016; Wardle et al. 2001).

### Soil properties

Soil abiotic properties were measured in all plots in both deer present and absent plots to aid in the interpretation of how changes to the ecosystem by sika deer influence insect herbivory. There was a litter layer approximately 3 cm deep on the surface of the soil that was removed from the soil samples, along with large pieces of leaf and woody litter, prior to analysis. Soil pH and conductivity were measured on a homogenized subsample of soil taken from each of the four corners of each plot in the field to a depth of 10 cm (pH meter Model#: B-71X and conductivity meter Model#: B-771, Horiba Scientific, Kyoto, Japan). Additional soil was sampled at the corner of each plot to a depth of 10 cm using a hand trowel; samples were bulked and homogenized per plot. Soil samples were stored frozen (−18 °C) until they were thawed and passed through a 4-mm mesh sieve to remove plant matter and stones and used in further analyses. Soil moisture was determined on a subsample of soil collected from each plot after drying (105 °C, 24 h). Soil organic matter (SOM) content was determined after combustion in a muffle furnace (550 °C, 4 h) on an additional subsample of soil from each plot. A further subsample of fresh soil (5 g fresh weight) was extracted with 50 ml 1 M KCl after shaking for 1 h, and extracts were frozen until analyzed for NO_3_-N, NH_4_-N, and PO_4_-P by colorimetry on an AutoAnalyser III (SEAL Analytical, Kontram OmniProcess AB, Solna, Sweden).

### Forest stand and understory plant community

To characterize the effect of deer presence versus absence on the understory plant community, the total cover of each plant species present in each plot was assessed using the Braun-Blanquet method (Braun-Blanquet et al. 1932). Since tree diversity and abundance could affect the understory vegetation (e.g., light availability, allelopathic effects), we characterized the tree community within the experimental area within a 5 m radius of each plot to ensure that all trees in and around all the plots were taken into account. We measured the diameter at breast height (DBH) of all trees and the canopy openness by taking five pictures of the canopy above each plot. We also measured the ground cover around each plot by taking four pictures around each plot from a 1 m distance to each side. The pictures of the canopy and ground were analyzed using the software ImageJ (see Online Resource 1).

### Plant traits, leaf damage and herbivory

**Fig. 2:**
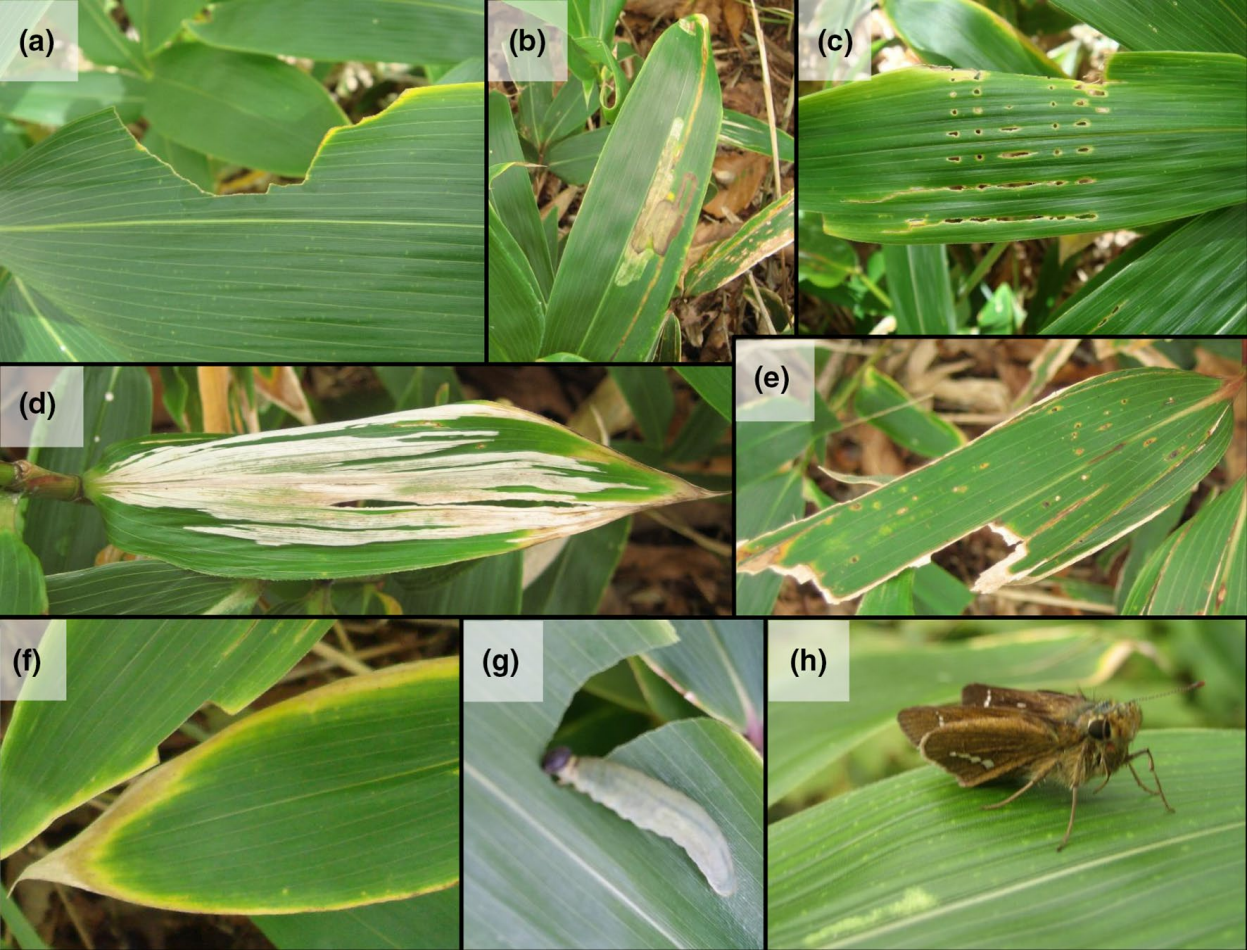
Types of herbivory and leaf damage to *Sasa palmata* observed in the study area. The different types of herbivory/damage observed on *Sasa palmata* leaves in this study: **a** chewing, **b** mining, **c** early chewing, **d** whitening, **e** indefinable damage, and **f** senescence. During the preliminary inspection we found likely herbivores on *Sasa palmata* such as a **g** caterpillar and a **h** moth. Color version of this figure is available online

We chose to conduct our study on *S. palmata* because it is the dominant understory plant in this system (Uchida et al. 2008). During preliminary inspection of herbivory on *S. palmata* leaves in the area of study, we observed three types of herbivory that were frequent, easy to identify and originated from different insect herbivore feeding guilds (Fig. 2). These types of herbivory included chewing (either on edges on leaf blades or new edges most likely caused by Lepidoptera larvae (Fig. 2g, h) or Orthoptera; hereafter: chewing; Fig. 2a) and mines within leaves caused by leaf miners (the larvae of Lepidoptera, Hymenoptera or Diptera) (hereafter: mining; Fig. 2b). We also observed equidistant holes tangential to the leaf tip that were most likely caused by an unknown insect herbivore boring or chewing through the young unfolded leaf (hereafter: early chewing; Fig. 2c). We also estimated the proportions of leaves that had turned white [likely due to over-wintering damage (Ide 2004)] (hereafter: whitening; Fig. 2d), damage due to indefinable causes (see Fig. 2e) and leaf senescence (obvious start of drying leaf edges; hereafter: senescence; Fig. 2f).

Within each of the 24 plots we selected five *S. palmata* plants. In order to maximize the capture of spatial heterogeneity within each plot, we searched for suitable plants in each corner of each plot and at the center of each plot. Because we aimed to compare damaged and undamaged leaves on the same shoot, we chose the shoots for insect herbivory estimation if they had at least one undamaged leaf (or only marginal leaf senescence as completely intact leaves were generally rare) and at least one leaf with chewing, early chewing or mining. If these criteria were not matched, we continued to inspect the next shoot, regardless of size and leaf number it possessed. For each selected shoot (hereafter: main shoot), we also identified side shoots, which were then also used (hereafter: side shoot). For each leaf on all selected shoots, we estimated the percentage of damage due to the causes described above. The survey was performed at the end of the growing season in order to assess the cumulative effect of each insect herbivory type (Shimazaki and Miyashita 2002).

To help elucidate possible causes of deer herbivory on *S. palmata*, subsequent changes to leaf traits and the effect of these trait changes on the insect herbivores, a number of plant traits were inventoried in each plot. We counted leaves on each main/side shoot and measured the height of the shoots. Because increasing leaf toughness can reduce herbivory rates (Lambers and Poorter 1992), we also measured toughness of one damaged (chewing, mining or both; early chewing was infrequent, but was used in some rare cases due to absence of chewing/mining) and one undamaged leave on each main shoot using a Dial Tension Gauge (Model #546-125, Mitutoyo Corporation, Japan). Care was taken that damaged and undamaged leaves had similar leaf area and that measurements were always taken in the middle of the leaf (three measurements between the veins and averaged). This procedure was chosen because a preliminary test revealed that toughness increased with leaf size and with distance from the leaf tip (Online Resource 2). Leaf toughness measurements were standardized by leaf area because toughness values per leaf area can inform if plants responded to abiotic/biotic differences between deer treatments.

We also measured the chlorophyll content in damaged and undamaged leaves (SPAD-502 portable chlorophyll meter, Konika, Japan), because chlorophyll content is a proxy for leaf N content (Chang and Robison 2003), and higher leaf N content can lead to increased insect herbivory (Lambers and Poorter 1992). On the five main shoots within each plot three chlorophyll measurements were always taken on the damaged/undamaged leaves closest to the ground. Preliminary measurements showed no considerable variation of chlorophyll content due to leaf size (Online Resource 2) and the three measurements done on each of the undamaged leaves were always done at an increasing distance from the leaf base. Because we aimed to maximize detectability of changes due to deer herbivory, measurements on damaged leaves were taken in close proximity to the damage. In some rare cases the second and third measurement had to be performed on another damaged leaf because there was not enough leaf area left for more than one or two measurements.

Finally, the leaves on which the toughness measurements were taken were detached from each plant with a scissor and brought back to the field station where their fresh weights were measured. Pictures of each single leaf were taken (Olympus, Pen PL 1) and the leaf area calculated (mm^2^) using the ImageJ software (see Stephan et al. 2015 for precise description). Leaves were left to dry at room temperature (22 °C), the dry weight was measured, and specific leaf area (SLA) and leaf dry matter content (LDMC) were calculated. Leaves from each plot were bulked into damaged and undamaged treatments and then ground in a Wiley Mill (Model #: 3383-L40, Thomas Scientific, Swedesboro, New Jersey, USA). A subsample of 0.05 g leaf litter was extracted in 20 ml 50% analysis-grade methanol and shaken for 1 h. A sub-aliquot of this subsample was analyzed for total polyphenols using the Prussian Blue technique with a catechin standard (Stern et al. 1996). Total polyphenol concentration was measured because total polyphenols can impact upon insect herbivory (Coley et al. 1985).

### Calculations and data analysis

Response variables of count data (except standardized count data, see below) were analyzed with generalized linear mixed models (GLMMs) with Poisson distributions and a log link function, while the remaining data were analyzed either with linear models (LM) or linear mixed models (LMM). The LMs and LMMs were validated visually (Zuur et al. 2010) leading to transformations of some response variables (Tables 1, 2). In the LMMs, nested Gaussian random factors accounted for the hierarchical data structure (e.g., plant nested within plot), while in all GLMMs random factors (e.g., shoot nested within plant nested within plot) were also taken into account. Type-III analysis-of-deviance tables with Wald Chi-square tests were utilized during the backward selection procedures. The litter cover showed no variation, as it was always 100% in the deer-absent treatment. We therefore compiled a table with the frequency of each litter cover for the five estimated percentages of cover of each treatment and analyzed it with Fisher’s exact test. In six of the absent and eight of the deer-present plots the PO_4_-P concentrations were below detection limit and both deer treatments were therefore compared with the Kolmogorov–Smirnov test (ks test).

**Table 1:** Effect of the presence or absence of deer on abiotic soil characteristics (models 1–6) and the vegetation within and around each plot (models 7–11) using analysis-of-deviance (Type III test)

Model		Response variable	Model type	Explanatory variable	*F* value/*χ* ^*2*^	*df*	*p* value	Deer exclosure
Abiotic soil properties	1	pH	LM	Deer treatment	0.7	1	0.403	→
				Residuals		22		
	2	Conductivity	LM	Deer treatment	2.7	1	0.108	→
				Residuals		22		
	3	Soil water content	LM	Deer treatment	21.8	1	<0.001	↑
				Residuals		22		
	4	Soil organic matter	LM	Deer treatment	13.7	1	0.001	↑
				Residuals		22		
	5	NH_4_-N	LM	Deer treatment	17.5	1	<0.001	↑
				Residuals		22		
	6	NO_3_-N (log)	LM	Deer treatment	0.0	1	0.993	→
				Residuals		22		
Vegetation	7	Canopy openness [%] (sqrt)	LM	Deer treatment	2.0	1	0.161	→
				Residuals		22		
	8	Ground cover [%]	LM	Deer treatment	7.1	1	0.013	↓
				Residuals		22		
	9	Understory plant abundance	GLMM (plot)	Deer treatment	0.0	1	0.947	→
	10	Understory plant richness	GLMM (plot)	Deer treatment	29.0	1	<0.001	↓
	11	Plant diversity [Shannon] (sqrt)	LM	Deer treatment	17.7	1	<0.001	↓
				Residuals		22		

Where applicable, transformations to meet requirements for the LMs are given behind the response variable in parentheses (*sqrt* square root) and the random factor for mixed models is stated in brackets. The last column indicates the direction of the response variable due to deer exclosure (↑/↓ indicates strong increase/decrease, → indicates no change)

*LM* linear model, *GLMM* general linear mixed model

**Table 2:** Effect of the presence or absence of deer on the traits of *Sasa palmata* using analysis-of-deviance (Type III test); (models 12–19; due to no differences among insect feeding guild herbivory; specifies either chewing/mining/both or undamaged leaves), and on the insect herbivory these plants experienced (models 20–22; chewing, mining or early chewing)

Model		Response variable	Model type	Explanatory variable	*χ* ^*2*^	*df*	*p* value	Deer exclosure
Plant and leaf traits	12	Shoot height	LMM (plot/plant)	Deer treatment	94.9	1	<0.001	↑
	13	Leaves per shoot	GLMM (plot/plant/shoot)	Deer treatment	18.1	1	<0.001	↑
	14	Leaf area	LMM (plot/plant)	Herbivory	7.8	1	0.005	↑
				Deer treatment	86.6	1	<0.001	
				Deer treatment × herbivory	0.4	1	0.495	
	15	Leaf toughness	LMM (plot/plant)	Herbivory	6.2	1	0.012	↓
				Deer treatment	8.8	1	0.002	
				Deer treatment × herbivory	3.1	1	0.075	
	16	Leaf toughness* leaf area (log)	LMM (plot/plant)	Herbivory	6.6	1	0.009	↑
				Deer treatment	117.1	1	<0.001	
				Deer treatment × herbivory	0.0	1	0.964	
	17	Chlorophyll	LMM (plot/plant)	Herbivory	178.3	1	<0.001	→
				Deer treatment	2.1	1	0.138	
				Deer treatment × herbivory	0.0	1	0.878	
	18	Specific leaf area (sqrt)	LMM (plot/plant)	Herbivory	0.2	1	0.648	↘
				Deer treatment	2.8	1	0.091	
				Deer treatment × herbivory	1.6	1	0.194	
	19	Leaf dry matter content (log)	LMM (plot/plant)	Herbivory	4.3	1	0.037	↘
				Deer treatment	3.2	1	0.073	
				Deer treatment × herbivory	1.3	1	0.249	
	20	Total polyphenols	LMM (plot)	Herbivory	1.2	1	0.267	↑
				Deer treatment	12.7	1	<0.001	
				Deer treatment × herbivory	2.3	1	0.123	
Insect herbivory	21	Incidence per leaf (log)	LMM (plot)	Feeding guild	9.4	2	0.008	→
				Deer treatment	0.0	1	0.883	
				Deer treatment × feeding guild	2.2	2	0.327	
	22	Herbivory per leaf (log)	LMM (plot)	Feeding guild	47.1	2	<0.001	↑
				Deer treatment	5.1	1	0.023	
				Deer treatment × feeding guild	1.6	2	0.443	
	23	Incidence per leaf per area (log)	LMM (plot)	Feeding guild	7.5	2	0.023	↓
				Deer treatment	4.4	1	0.034	
				Deer treatment × feeding guild	2.4	2	0.300	
	24	Herbivory per leaf per area (log)	LMM (plot)	Feeding guild	42.1	2	<0.001	↓
				Deer treatment	25.4	1	<0.001	
				Deer treatment × feeding guild	1.7	2	0.408	

If possible, non-significant terms were removed stepwise from the final model (non-bold). Where applicable, transformations to meet requirements for the LMMs are given behind the response variable in parentheses (*sqrt* square root) and random factors are stated in brackets (“/” indicates “nested within”). The last column indicates the direction of the response variable due to deer exclosure (↑/↓ indicates strong increase/decrease; ↗/↘ indicates tendency of increase/decrease)

*LMM* linear mixed model, *GLMM* general linear mixed model

The estimations of insect herbivory (see Online Resource 3 for original values and other damage types) were analyzed in several ways. We aimed to quantify insect abundances, the extent of their feeding and quantify the damage individual *S. palmata* plants experienced on a per area basis. Therefore, we performed a number of calculations:

1. We calculated the number of incidences of each type of herbivory (i.e., chewing, mining, early chewing) on a plant and divided it by the number of shoots and the number of leaves on a shoot. Therefore, these incidences are measured on a per leaf-basis and can be compared among deer treatment and herbivory type; hereafter incidence per leaf.
2. To estimate how much leaf area was consumed per leaf, we calculated the insect herbivory in cm^2^; hereafter herbivory per leaf. To account for leaf size differences (Online Resource 4), herbivory per leaf was calculated by using the mean of all undamaged leaves (similar size as damaged; see above) of each plot multiplied with the estimated percentage herbivory of each leaf. Therefore, the total leaf area consumed between herbivory types and deer treatments could be compared.
3. The incidence per leaf was further standardized by dividing it by the estimated percentage cover of *S. palmata* within each plot; hereafter incidence per leaf per area. We did not standardize by leaf area resulting in incidences per cm^2^ because the major leaf area difference was between deer-absent/present treatments and long distance host plant choices by insects are likely to occur between these deer-present/absent habitats.
4. Further, we calculated the herbivory per leaf per area by dividing the percentage damage (standardized by leaf area) by the number of leaves on a shoot and the estimated percentage cover of *S. palmata* within each plot; hereafter herbivory per leaf per area. This measure can be used as an indicator of how resistant/suitable the same leaf area of *S. palmata* plants in deer absence/presence are to insect herbivory.

By performing calculations (3) and (4), we accounted for the dilution of herbivory (i.e., the feeding site selection of Orthoptera or oviposition site selection by adult Lepidoptera), which should lead to variation in the presence of chewing caterpillars and leaf miners due to different available cumulative leaf area per 1 m^2^ plot area. Therefore, these measurements allow for upscaling of insect damage to the ecosystem level.

In order to investigate the direct and indirect relationships between the soil properties, the understory plant community, the traits of *S. palmata* and the insect herbivory, we built a partial least square path model (PLS-PM). This type of structural equation model is robust, does not rely on normal distribution or independence of data, and can be performed with limited data (Chin and Dibbern 2010). Further, it allows for the calculation of latent variables and the paths between them, and therefore avoids an unnecessarily confusing number of paths (Majdi et al. 2014, Musseau et al. 2015). The creation of latent variables from measured variables (e.g., the latent variable “soil properties” consisted of the measured variables SOM, water content, NH_4_-N) allows for broad conclusions to be reached about the effect that interrelated variables have on different sets of reflective variables. Most importantly, such PLS-PMs allow for comparison of paths between the two separate models for deer absence and presence. For more details on the construction of the PLS-PM, please see Online Resource 5.

Analyses and figures were generated with the R software (R Core Team 2015) with the packages *lme4* (Bates et al. 2014), *car* (Fox and Weisberg 2011), *cairo* (Urbanek and Horner 2014), *multcomp* (Hothorn et al. 2008), *paircompviz* (Burda 2013), and *vegan* (Oksanen et al. 2012). The PLS-PM was built using the package *plspm* (Sánchez et al. 2015).

## Results

### Soil properties

Soil gravimetric water content, SOM and NH_4_-N were all higher in deer-absent plots, while other soil properties such as pH, conductivity and NO_3_-N and PO_4_-P concentrations were not affected by the deer treatments (Table 1; Online Resource 6; ks test for PO_4_-P: *D* = 0.25, *p* = 0.847).

### Forest stand and understory plant community measurements

A number of forest stand and understory plant community parameters were affected by deer absence (Table 1; Online Resource 7). Understory ground cover with vegetation was higher in deer presence, while the litter cover tended to be higher in deer absence. Although understory plant abundance remained constant, the understory plant community richness and diversity (Shannon Index) (dominant species, besides *S. palmata*, were *Aster ageratoides, Carex alopecuroides* var *chlorostachys*, and *Thelypteris nipponica*) were higher when deer were present (Online Resource 8). We did not find differences in the canopy openness between the different deer treatments, but tree abundance (consisting of *Betula platyphylla* var *japonica*, *Euonymus sieboldianus* var *sanguineus*, *Malus toringo*, *Q. crispula*, *Ulmus davidiana* var *japonica*), diversity and evenness were higher when deer were absent (Online Resource 9) and the DBH was also higher in the deer-present side and lowest for *M. toringo* (Online Resource 10).

### Plant/leaf traits and leaf damage/herbivory

Nearly all *S. palmata* plant and leaf characteristics were affected by deer presence versus absence and/or insect herbivory with no significant interactions (Table 2; Online Resource 11). In the deer-absent treatment, *S. palmata* shoots were around 40 cm taller and had approximately two more leaves that were twice as large compared to plants in the deer-present treatment. Leaves were around twice as tough (i.e., relative measure) in deer absence and SLA and LDMC tended to be lower compared to the deer-present treatment. In contrast, total polyphenol concentrations were higher in the deer-absent treatment. While we did not detect differences in leaf chlorophyll content, leaf toughness (i.e., standardized by leaf area) was higher in deer presence. Leaves with insect herbivory had lower area, were generally tougher (i.e., standardized by leaf area), exhibited lower chlorophyll content, and had higher LDMC compared to undamaged leaves.

**Fig. 3:**
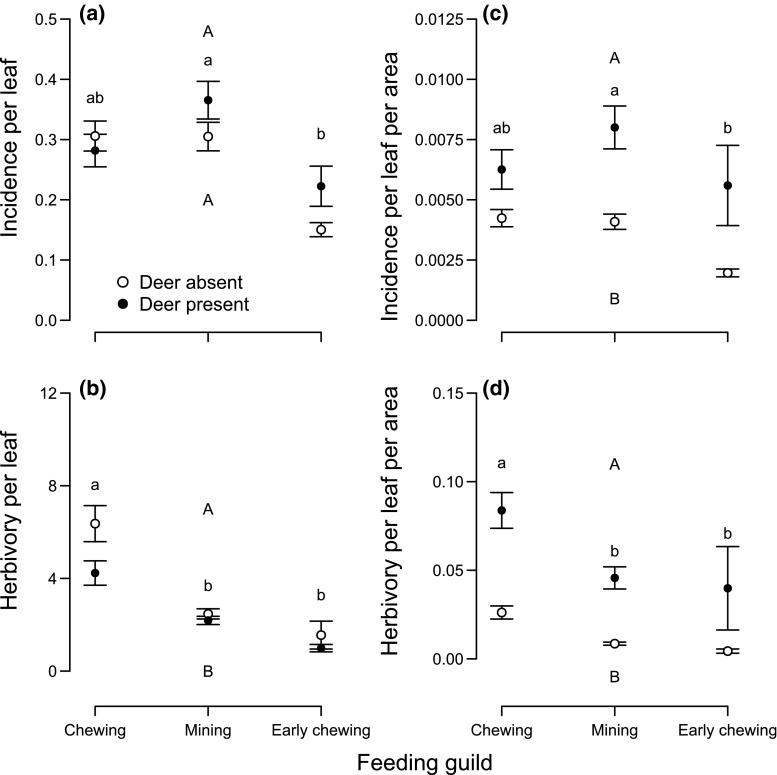
Figure depicting how often any *Sasa palmata* leaf experienced **a** herbivory (i.e., incidence) and **b** how much leaf area was consumed in cm^2^ (i.e., herbivory) by all three insect-feeding guilds with regard to deer treatment and how often each plant experienced **c** herbivory (i.e., incidence) and **d** the herbivory after accounting for the dilution of herbivory due to larger/more abundant host plants. *Different upper case letters* indicate significant differences in overall insect herbivory between deer treatments and *different lower case letters* indicate differences between individual insect-feeding guilds across both deer-present and deer-absence plots (*p* < 0.05; Tukey contrast). All values are mean ± SE

There were some significant differences in insect herbivory on *S. palmata* plants generated by the absence versus presence of sika deer (Table 2; Fig. 3). Incidence per leaf generated by the three insect-feeding guilds (Fig. 3a) and most other types of damage (Online Resource 3) occurred with the same frequency in both deer present and absent plots. Insect herbivory per unit leaf area was overall higher across all three insect-feeding guilds in the deer-absent treatment (Fig. 3b). In contrast, insect herbivory incidence per leaf by % cover (Fig. 3c) and herbivory per leaf per plot (Fig. 3d) were overall generally higher in the deer-present treatment. Regardless of deer treatment and dilution of herbivory due to more/larger resources, mining was the most frequently observed herbivory type and chewing was the most severe.

### The impact of deer exclosure on ecosystem functioning

**Fig. 4:**
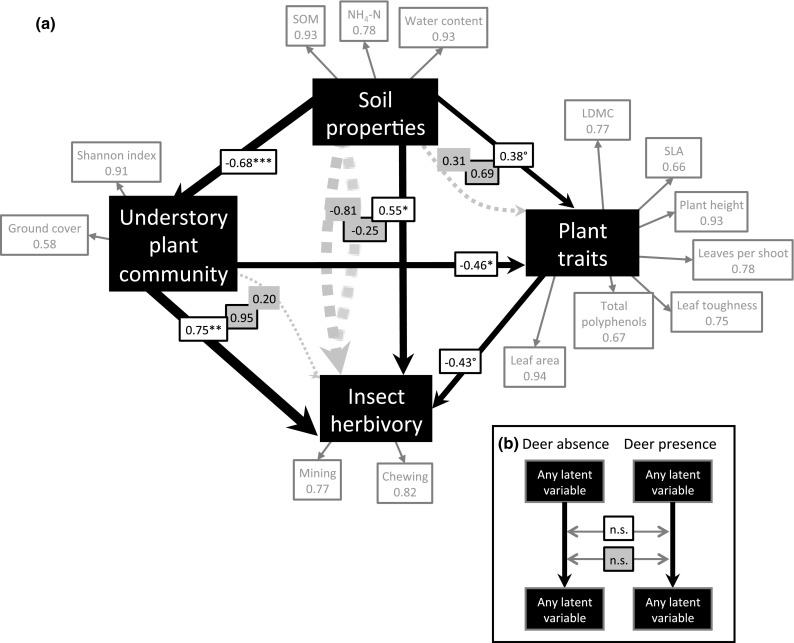
**a** Partial least square path model summarizing the functioning of the ecosystem. The reflective latent variables (*black boxes*) are linked with several measured indicators (*grey boxes* indicating contribution of each measured indicator to the path coefficients). *Black arrows* and *white boxes* indicate direct paths between variables, *grey dashed arrows* and *grey unframed boxes* indicate the indirect effects via other paths, and *grey boxes with black frames* illustrate the total effect (i.e., combined direct and indirect effects). *Arrow* widths are proportional to strength of the coefficient; ***p < 0.001; **p < 0.01; *p < 0.05; °*p* < 0.06. Performing **b** separate models for deer presence/absence and comparing the bootstrapped direct and total effects revealed no significant differences between the models. Except polyphenols, all trait values are means from several plants/shoots/leaves within a plot. Leaf dry matter content (LDMC) and specific leaf area (SLA) were multiplied by negative one to meet the requirement of unidimensionality of indicators. *SOM* soil organic matter

The final PLS-PM (Fig. 4) revealed that soil properties were overall strong drivers of the understory plant community (path coefficient: −0.68). Due to the use of unidimensional reflective indicators in the model, changes in indicators can be linked directly to changes in other indicators (Online Resource 5). For example, higher NH_4_-N was associated with lower plant diversity. Further, soil properties were important direct drivers of insect herbivory (e.g., higher SOM led to higher herbivory by chewing/mining insects; 0.55), and modest direct drivers of plant traits (0.38). However, soil properties also indirectly positively influenced plant traits via the plant community (0.31) resulting in a strong total effect (0.69). The indirect effect of soil properties on insect herbivory via plant traits and understory plant community was very strong and negative (−0.81), resulting in a weak net negative effect (−0.25). The understory plant community had a strong positive direct effect on insect herbivory (0.75), which was strengthened by a positive (albeit weak) indirect effect via plant traits (0.20; 0.95 total effect). Plant traits were negatively affected by the plant community (e.g., lowered understory plant diversity, which led to taller plants, but lower LDMC and SLA; −0.46). Plant traits had a negative effect on insect herbivory (−0.43). Comparing the paths from separate PLS-PMs for the deer absent and present treatments revealed that neither the direct, nor the total effects significantly differed between models (Fig. 4b; Online Resource 12).

## Discussion

In this study we investigated how the absence versus presence of sika deer altered soil properties, the understory plant community, plant traits and insect herbivory, as well as the functional relationships between them. We found that the absence of sika deer generally positively impacted upon *S. palmata* traits, decreased the understory plant diversity and altered soil abiotic properties. Although insect herbivory incidence per leaf was not different between deer treatments, insect herbivory per leaf (i.e., leaf area consumed) was highest in the deer-absent treatment. This may indicate higher herbivore abundance in deer absence. In contrast, we observed higher insect herbivory incidence and herbivory per plant per area in the deer-present plots, which indicates that, on a per area basis, *S. palmata* plants experienced greater herbivory pressure in deer presence. Our path model revealed that despite direct and indirect effects of soil properties, the understory plant community and plant traits on insect herbivory, functional relationships between these ecosystem components were not altered by deer presence versus absence. These results provide evidence that deer can alter ecosystem properties and interactions between different trophic levels in this system, but not necessarily the relationships between them. Below we explore how these results advance our understanding of deer herbivory-initiated trophic cascades that may have consequences for the functioning of forested ecosystems.

In support of our first hypothesis, insect herbivory per leaf was higher when deer were absent (Fig. 3b). However, contrary to our expectation, leaves in deer absence were tougher (i.e., relative measure) with higher concentrations of defensive polyphenol compounds. Interestingly, insect herbivory likely increased leaf toughness (i.e., standardized by leaf area), which is a finding surprisingly not well supported (but see Robison and Raffa 1997; Fornoff and Gross 2014). Contrary to our expectation, increased consumption by insect herbivores likely cannot explain the observed increased herbivory per leaf when deer were absent. Instead, it seems most probable that an increase in insect herbivore abundance generated this result (Castagneyrol et al. 2013), although we did not measure insect abundance directly. This interpretation is further supported by the general observation that better defended plants increase insect feeding time (Feeny 1970), This means that the same insect abundance in deer absence and presence would generate less herbivory in deer presence with increased leaf toughness and polyphenol concentrations (Online Resource 11). Increased insect abundance in deer absence therefore may have even compensated for such a likely prolonged feeding time, leading to higher herbivory in deer absence plots. The amount of leaf area consumed (i.e., herbivory per leaf) therefore seems a more reliable approximation of insect abundance (Kim 2014) than herbivory incidence per leaf, which was not different between deer treatments (Fig. 3a). Further, although the buildup of larger populations of less mobile insect herbivores may also contribute to the observed increase in insect herbivory per leaf, the main drivers were most likely higher preferences for more apparent food sources (i.e., taller plants, more leaves, higher cover) in the deer-absent treatment compared to the deer-present treatment (Whitham 1978; Gripenberg et al. 2010). This is supported by the work of Tabuchi et al. (2010), who showed that the presence of sika deer decreased ovipositing by the gall midge *P. uedai* on *Sasa* species. Finally the higher herbivory per leaf may additionally be attributed to decreased competition among insects, due to higher resource availability when deer are not consuming large quantities of *S. palmata* (Aparicio et al. 2015).

Our second hypothesis was supported because both insect herbivory incidence per leaf per area and herbivory per leaf per area (i.e., accounting for the dilution of herbivory due to more abundant and larger resources) were higher in deer presence (Fig. 3c, d). These findings are indicative of a number of things. First, deer presence has created a more heterogeneous habitat through increased plant diversity (Table 1; Online Resource 7), which might make host plant detection more difficult for insect herbivores (Coll and Bottrell 1994; Hambäck et al. 2014). Therefore, in deer absence insect herbivory was diluted, while in deer presence fewer host plants led to more insects per individual plant (so-called insect load, e.g., Otway et al. 2005). This view, related to classical theories on how insect herbivores respond to host plant monocultures (Root 1973), is supported by the increased vertical vegetation complexity (i.e., increased ground cover) in the deer-present plots and possibly further decreased host plant encounters. From the plant perspective, this means that deer are directly feeding upon *S. palmata* and also indirectly increasing insect herbivory on individual *S. palmata* plants. Second, it supports our interpretation of higher insect herbivore abundances in deer absence. This is because, despite a strong dilution of herbivory in deer absence, insect herbivory per leaf was still greater when deer were absent.

There was no support for our third hypothesis because contrasting insect-feeding guilds did not damage *S. palmata* differently depending upon deer presence or absence. Instead, insect herbivory per leaf (and per unit area) by chewing insects was greater than mining and early chewing regardless of deer presence or absence. This is in contrast to findings by Barrett and Stiling (2007), who showed that the presence of deer positively affected leaf miners and proposed that leaf miners, a specialist herbivore, were more sensitive to vegetation patch size dynamics than generalist herbivores (e.g., chewing insects). Given that in our system the understory consisted predominantly of *S. palmata* (Online Resource 8), it is likely that the high abundance of this resource reduced such patch dynamics, thereby resulting in the similar responses across contrasting insect-feeding guilds. Furthermore, having very different and/or unpalatable plants in the vicinity (i.e., *A. ageratoides*) and greater understory plant species richness near *S. palmata* plants growing in the deer-present plots may have increased the associational resistance (i.e., associations with certain plants that decrease vulnerability to/detection by herbivores) against all insect-feeding guilds equally (Barbosa et al. 2009; Muiruri et al. 2015). Alternatively, these findings may indicate that resource utilization of the insect guilds attacking *S. palmata* may be the same. This could mean that changes to deer population densities in the region will continue to affect herbivorous insects similarly, allowing for broad-scale predictions of the impacts of an expanding deer population on ecological processes that are driven by insect herbivores from different feeding guilds.

Our fourth hypothesis was supported in so far as deer generated changes to soil properties, the understory plant community and plant traits, which in turn created indirect impacts on insect herbivory (Fig. 4a). According to the path model, a more diverse understory plant community and higher cover of *S. palmata* led to increased insect herbivory. This supports our interpretation that higher plant diversity dilutes insect herbivory and therefore increases herbivory on individual plants, while higher cover of a target host plant (i.e., *S. palmata*) generally increased insect herbivore attraction/abundance. It also compliments other work that has demonstrated deer-induced changes to plant community composition can lead to impacts on the insect community (Kanda et al. 2005). In contrast, the effect of plant traits was detrimental to insect herbivory, indicating that deer can indirectly alter plant traits, which leads to changes in insect herbivory (Takagi and Miyashita 2015). Furthermore, the path model revealed that soil properties had a net negative (albeit weak) effect on insect herbivory. This was likely because although insect herbivory was positively affected by the direct effect of soil properties, the positive effect was overridden by the negative indirect effects of soil properties as mediated by plant traits and the understory plant community. This corresponds to the observed higher insect herbivory per plant per area observed when deer were present (Fig. 3d). Additionally, soil properties exerted a positive direct effect on plant traits that was further strengthened by indirect effects mediated via the understory plant community. This supports other work showing that deer can alter the plant traits via soil-mediated effects (Kardol et al. 2014), as changes in the soil parameters were clearly driven by deer presence.

Finally, and in contrast to the second part of our fourth hypothesis, we found that none of the paths differed significantly between the two models. This indicates that the functional relationships between these ecosystem properties were unaffected by deer presence versus absence in this system (Fig. 4b; Online Resource 12). For example, plant traits had a negative effect on insect herbivory. However, the differences in plant traits within deer-present and within deer-absent treatments affected insect herbivory similarly. This suggests ecosystems may function the same with or without deer, despite both direct and indirect alterations to a number of ecosystem processes; see above. While this functional relationship is a relative measure of the relationships among the ecosystem components, it does not inform on the absolute effect of deer presence versus absence. For example, if deers cause plants to become more conservative in their trait expression (i.e., phenotypic plasticity), insect herbivory will likely decrease (Shikata et al. 2013). This could have negative impacts on insect population dynamics, which could alter nutrient cycling (i.e., insect frass returning nutrients to the system). Therefore, although overall functional relationships might be unaffected, net balance of nutrient cycling could decrease with deer presence.

Deer populations are increasing on a global level (Côté et al. 2004), with such increases exacerbated as the global climate becomes milder (Grotan et al. 2005). We showed that the presence of deer caused significant redistributions of organic matter and nutrients, impacting upon plant (Den Herder et al. 2004) and leaf traits (Shikata et al. 2013; Shimazaki and Miyashita 2002), which led to very different phenotypes (i.e., trait expression) of *S. palmata*. These changes to nutrient availability and plant traits led to increased insect herbivory incidence and herbivory per plant per area when deer were present, while when deer were absent, *S. palmata* could compensate for insect herbivory, likely due to more favorable conditions. However, caution must be taken when extrapolating our results to other systems, where increased understory diversity could generate different interaction effects. For example, increasing understory plant diversity may lead to higher (Stephan et al. 2016) or lower (Suominen et al. 2008) abundance and a community shift of predacious arthropods and ultimately decrease or increase insect herbivory. Furthermore, the impact of deer grazing on insect herbivory might be altered according to habitat resource availability (Vesterlund et al. 2012). In addition, interactions between an even greater number of trophic levels (e.g., deer, plants, insects, predators) should be considered (Bailey and Whitham 2003). Taken together, these results demonstrate the importance of considering how deer presence can alter the soil properties, understory plant community composition and plant traits, thereby impacting upon ecosystem processes (Côté et al. 2004). Even though our model demonstrated that the functional relationships between the measured ecosystem processes were not altered by deer presence in this system, the net balance of ecosystem processes (e.g., nutrient flux, primary productivity) might be increased or decreased. Finally, our results shed light on the necessity of examining how increasing deer populations affect interactions between different ecosystem properties when making predictions about ecosystem function.

## Electronic supplementary material

Below is the link to the electronic supplementary material.

Supplementary material 1 (DOCX 605 kb)
