# Supplementary material for: Long-term deer exclosure alters soil properties, plant traits, understory plant community and insect herbivory, but not the functional relationships among them
Source: Oecologia. 2017 Jul 1;184(3):685–99. doi: 10.1007/s00442-017-3895-3 (PMC5511341; doi:10.1007/s00442-017-3895-3)
Supplement: Supplementary file 1 — Supplementary material 1 (DOCX 605 kb) [file 442_2017_3895_MOESM1_ESM.docx]

**Long-term deer exclosure alters soil properties, plant traits, understory plant community and insect herbivory, but not the functional relationships among them**

Jörg G. Stephan, Fereshteh Pourazari, Kristina Tattersdill, Takuya Kobayashi, Keita Nishizawa and Jonathan R. De Long

**Online Resource 1: Measuring ground cover and canopy openness in the deer absent and deer present treatments using ImageJ**

Ground cover: On all four sides of each plot we stood 1 meter from the plot edge, outstretched the arm and made a picture of the ground (Canon IXUS 60). In ImageJ we made use of the *Threshold Colour* plugin and set the minimum hue to 50, activated threshold (leaving only the green part in the picture), converted the image type to 8-bit and measured the area. Because all pictures were taken at the same height above the ground (1.50 m) the area (percentage of all pixels) reflects the cover of green in the picture. All four measurements from all four sides of a plot were averaged to yield one value per plot.


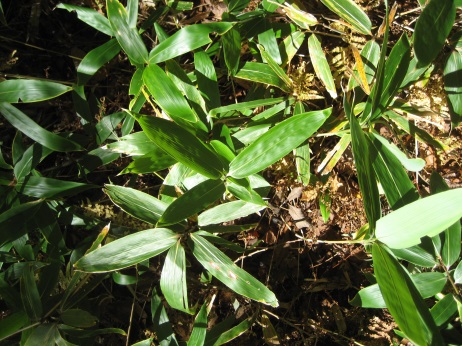

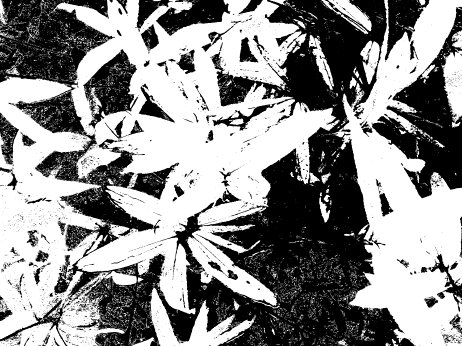


Canopy openness: Standing on the plot edge closest to the fence, we took one picture of the canopy by holding the camera directly above the head, and four pictures by holding it at a 45° angle on each side of the head. These pictures were analyzed in the same way as the ground cover pictures only that everything except the green part was measured (hue between 0 and 100). All five measurements per plot were averaged to yield one value per plot. The procedure was automated to be applied to all pictures by inserting the code of both procedures in the batch function in ImageJ.


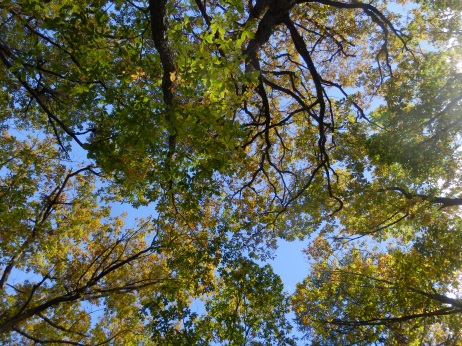

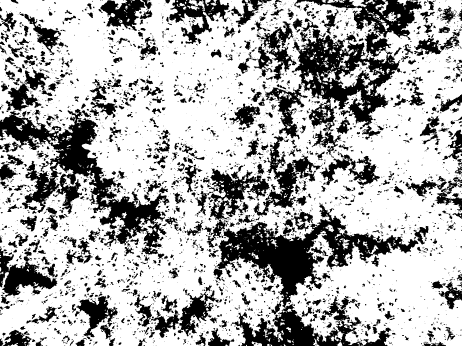


**Long-term deer exclosure alters soil properties, plant traits, understory plant community and insect herbivory, but not the functional relationships among them**

Jörg G. Stephan, Fereshteh Pourazari, Kristina Tattersdill, Takuya Kobayashi, Keita Nishizawa and Jonathan R. De Long

**Online Resource 2: Leaf toughness and chlorophyll content depending on location on leaf and leaf size**


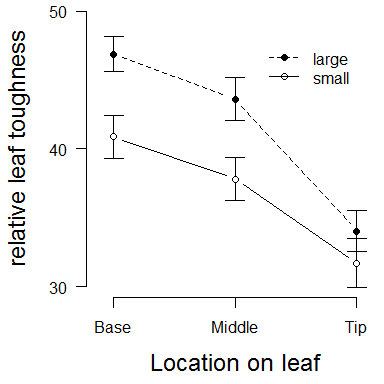


Mean (± SE) of relative leaf toughness of ten small (tip to base: ~10 cm) and ten large (~20 cm) *Sasa palmata* leaves with three measurements at each location on each leaf. The size of the leaf (LMM with leaf as random factor after removal of non-significant interaction: χ^2^= 6.9, p = 0.008) and the position along the leaf midrib (χ^2^= 105.5, p < 0.001) significantly affected the leaf toughness. Contrarily, the SPAD (portable chlorophyll meter) values were independent from the leaf size (mean ± SE: large: 42.1 ±0.7; small: 41.5 ± 0.8; Kolmogorov-Smirnov test: D = 0.3; p = 0.759).

**Long-term deer exclosure alters soil properties, plant traits, understory plant community and insect herbivory, but not the functional relationships among them**

Jörg G. Stephan, Fereshteh Pourazari, Kristina Tattersdill, Takuya Kobayashi, Keita Nishizawa and Jonathan R. De Long

**Online Resource 3: Original and partly standardized values of herbivory and other types of damage on *Sasa palmata***


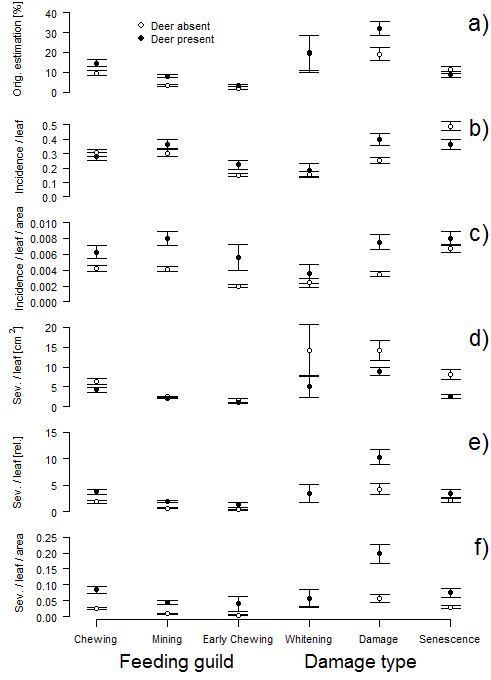


For all three insect feeding guilds, the herbivory and other damage types (hereafter: severity specifies both damage types) shown are (a) the original estimated percent per leaf, (b) the incidences per leaf, (c) the incidences per leaf and per ground cover of *S. palmata* in each plot, (d) the severity per leaf in cm^2^ leaf area consumed (calculated using the mean leaf area of all undamaged leaves on a plot; see Online Resource 4), (e) the relative severity (original values divided by leaf area), and the severity per leaf per plot (relative severity further divided by cover in each plot). All values are mean ± SE.

**Long-term deer exclosure alters soil properties, plant traits, understory plant community and insect herbivory, but not the functional relationships among them**

Jörg G. Stephan, Fereshteh Pourazari, Kristina Tattersdill, Takuya Kobayashi, Keita Nishizawa and Jonathan R. De Long


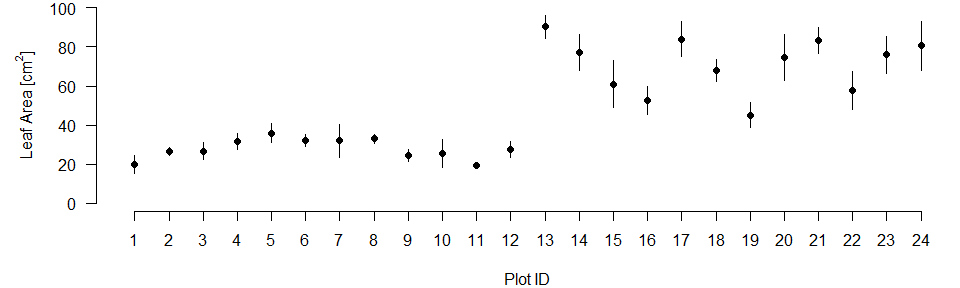
**Online Resource 4: Leaf area for each plot used to calculate absolute herbivory**

Variability (mean ± SE) of leaf area of undamaged *Sasa palmata* leaves (5 per plot) from 12 plots in deer present (1 to 12) and 12 plots in deer absent (13 to 24) treatments.

**Long-term deer exclosure alters soil properties, plant traits, understory plant community and insect herbivory, but not the functional relationships among them**

Jörg G. Stephan, Fereshteh Pourazari, Kristina Tattersdill, Takuya Kobayashi, Keita Nishizawa and Jonathan R. De Long

**Online Resource 5: Building and evaluation of partial least square path model**

We used a partial least square path model (PLS-PM) to investigate the direct and indirect relationships between soil characteristics, plant traits, plant community, and insect herbivory and to examine if these relationships were altered by deer exclosure (Sanchez et al. 2015). This type of structural equation model (SEM) is robust, does not rely on normal distribution or independence of data, and can be performed with limited data (Chin and Dibbern 2010). We decided to use a PLS-PM (versus covariance-based SEM) because we had a relatively small data set (16 measured variables, but only 12 replicates per deer treatment). Further, a PLS-PM allows for the calculation of latent variables (LVs), which is recommended for this type of model. This allows for comparison between the LVs and avoids an unnecessarily confusing number of paths. All variables were treated as reflective LVs, with each (dependent) indicator being regressed against the (independent) LV, meaning that changes in one variable will result in changes in the other. This is contrary to formative LVs, which represent a multiple regression using the indicators as independent variables. For example, species communities have been specified as reflective LV previously (Majdi et al. 2014). Indicators of reflective LVs need to be inspected based on loadings (i.e., the contribution of each measured indicator to the path coefficient; recommended to be higher than 0.7) and cross loadings (i.e., the contribution of each measured indicator to other path coefficients; should be highest for its own LV). LVs need to be evaluated based on: 1) average variance extracted (AVE) that describes the variance captured from indicators compared to variance due to measurement error (should be higher than 0.5) and 2) the composite reliability (Dillon-Goldstein’s rho) that indicates the unidimensionality of reflective indicators (should be larger than 0.7) (Vinzi et al. 2010). The paths between the LVs can be evaluated based on significance and effect size *f^2^* (based on the R^2^ of the dependent LV: *f^2^* = (R^2^_included_- R^2^_excluded_) / (1- R^2^_included_) (Chin and Dibbern 2010)), with values ≤ 0.15 indicating medium and values over 0.35 indicating strong effect sizes. The Goodness-of-Fit (GoF), which is a measure of the quality of the outer (indicators forming LVs) and inner model (LVs and paths) was also assessed (values of 0.7 are considered highly acceptable (Hair et al. 2011)).

Step 1: Selecting variables

In order to perform the PLS-PM, we calculated the means of all traits (except polyphenols because this measurement was already a plot aggregate value) for each plot resulting in values on plot level (n = 12 for each deer treatment). We used only the data rows of insect herbivory (except traits all other values were on plot level and it makes no sense to check for effects on zero herbivory) and selected the variables that responded to the deer treatment based on the previous analysis: soil properties (i.e., soil organic matter, ammonium, gravimetric water content), plant community (Shannon index, ground cover), insect herbivory (mining, chewing, early chewing), plant traits (leaf area, number of leaves per shoot, plant height, leaf toughness (relative), polyphenols, specific leaf area, leaf dry matter content, chlorophyll content).

Before creation of the PLS-PM, we recreated (lost during data collection) four measurements of leaf area (three for deer present; one for deer absent) using the leaf dry weight and the intercept and slope of a linear model between dry weight and leaf area. We also recreated two missing values for chlorophyll (one for each deer treatment) with the mean of all measurements. Because we used the values for deer absence plus presence we, if at all, lowered differences between deer treatments rather than falsely increasing them. Also, the aim of this PLS-PM was mainly to investigate the relationships between the LVs and both recreations strengthened the LVs. This is because for every value recreated, we gained seven values that could be included in the same LV (plus nine values for every recreated value that could be included in other latent variables).

Step 2: Building starting model and evaluating outer model

We first selected the path directions based on the general understanding of the abiotic and biotic drivers of insect herbivory (Fig. 1). We started by validating the outer model (indicators for LVs) by including all possible paths to the LV herbivory and the inner weighting scheme set to factorial, which ignores the directionality of the arrows among latent variables (Chin and Dibbern 2010). In order to fulfill the requirement of unidimensionality, we multiplied SLA and LDMC by minus one. Due to low loadings, we removed chlorophyll (0.46) and early chewing (0.42) from the model, which improved, the AVE for the LV plant traits from 0.57 to 0.63, the AVE for the LV insect herbivory from 0.44 to 0.63, and the composite reliability so that all LVs had values over 0.74. Also, the GoF of the model increased from 0.58 to 0.60 and all cross loadings were highest for their respective LV and validated using bootstrapping (5000 resampling’s)

Mean and standard deviations (SD) of loadings (contribution of each measured indicator to the path coefficient) after bootstrapping comparison of original values. SOM = soil organic matter, SLA = specific leaf area; LDMC = leaf dry matter content.

| Latent variable | Indicator | Original | Mean | SD |
| --- | --- | --- | --- | --- |
| Soil properties | Water content | 0.939 | 0.936 | 0.031 |
| Soil properties | SOM | 0.930 | 0.924 | 0.040 |
| Soil properties | NH_4_-N | 0.784 | 0.785 | 0.067 |
| Plant community | Ground cover | 0.584 | 0.563 | 0.204 |
| Plant community | Shannon index | 0.911 | 0.851 | 0.337 |
| Plant traits | Polyphenols | 0.672 | 0.67 | 0.116 |
| Plant traits | Plant height | 0.932 | 0.937 | 0.021 |
| Plant traits | Leaves per shoot | 0.78 | 0.775 | 0.089 |
| Plant traits | Leaf area | 0.942 | 0.945 | 0.012 |
| Plant traits | SLA*(-1) | 0.663 | 0.659 | 0.131 |
| Plant traits | LDMC*(-1) | 0.775 | 0.768 | 0.100 |
| Plant traits | Leaf toughness | 0.756 | 0.752 | 0.088 |
| Insect herbivory | Mining | 0.775 | 0.697 | 0.319 |
| Insect herbivory | Chewing | 0.827 | 0.767 | 0.330 |

Step 3: Evaluation of inner model

On this new model we then validated the inner model (i.e., LVs and paths) by specifying the path-weighting scheme, which accounts for both the strength and the direction of the paths in the structural model (Vinzi et al. 2010). Based on path significance and effect size, we kept the path from plant traits to insect herbivory (p = 0.058; *f^2^* = 0.91), and the path from soil properties to insect herbivory (p = 0.057; *f^2^* = 0.17) due to very large and medium effect size, respectively. All remaining paths had high coefficients and were significant. This model represents the final model and was further validated using bootstrapping (5000 resamplings).

Original, mean and standard deviations (SD) of direct and total path coefficients after bootstrapping.

| Paths | | | Direct effect | | | Total effect | | |
| --- | --- | --- | --- | --- | --- | --- | --- | --- |
|  |  |  | Original | Mean | SD | Original | Mean | SD |
| Soil properties | -> | Plant community | -0.683 | -0.648 | 0.253 | -0.683 | -0.648 | 0.253 |
| Soil properties | -> | Plant traits | 0.381 | 0.379 | 0.178 | 0.696 | 0.708 | 0.092 |
| Soil properties | -> | Insect herbivory | 0.557 | 0.496 | 0.376 | -0.259 | -0.254 | 0.229 |
| Plant community | -> | Plant traits | -0.462 | -0.441 | 0.255 | -0.462 | -0.441 | 0.255 |
| Plant community | -> | Insect herbivory | 0.751 | 0.705 | 0.415 | 0.952 | 0.852 | 0.442 |
| Plant traits | -> | Insect herbivory | -0.433 | -0.359 | 0.351 | -0.433 | -0.359 | 0.351 |

Step 4: Comparing models of deer absent and deer present

In order to compare the effect of deer exclosure on the paths between the LVs, we built separate models with the structure of the final model. Although it may be applicable to compare path coefficients directly (Musseau et al. 2015), we decided to evaluate the differences more thoroughly using bootstrapping followed by t-tests between coefficients of direct and total effects (indirect effects are not implemented in the package used).

**References**

Chin WW, Dibbern J (2010) How to Write Up and Report PLS Analyses. In: Vinzi VE, Chin

WW, Henseler J, Wang H (eds) Handbook of Partial Least Squares Concepts, Methods and

Applications. Springer, Heidelberg, pp 171–193

Hair JF, Ringle CM, Sarstedt M (2011) PLS-SEM: Indeed a Silver Bullet. J Mark Theory Pract

19:139–152. doi: 10.2753/MTP1069-6679190202

Majdi N, Boiché A, Traunspurger W, Lecerf A (2014) Predator effects on a detritus-based food

web are primarily mediated by non-trophic interactions. J Anim Ecol 83:953–962. doi:

10.1111/1365-2656.12189

Musseau C, Vincenzi S, Jesensek D, et al (2015) Direct and indirect effects of environmental

factors on dietary niches in size-structured populations of a wild salmonid. Ecosphere 6:1–15.

doi: 10.1890/ES15-00109.1

Sanchez G, Trinchera L, Russolillo G (2015) Tools for Partial Least Squares Path Modeling

(PLS-PM).

Vinzi VE, Trinchera L, Amato S (2010) PLS PathModeling: From Foundations to Recent

Developments and Open Issues forModel Assessment and Improvement. In: Handbook of Partial Least Squares. pp 171–193

**Long-term deer exclosure alters soil properties, plant traits, understory plant community and insect herbivory, but not the functional relationships among them**

Jörg G. Stephan, Fereshteh Pourazari, Kristina Tattersdill, Takuya Kobayashi, Keita Nishizawa and Jonathan R. De Long

**Online Resource 6: Soil properties depending on deer treatment**


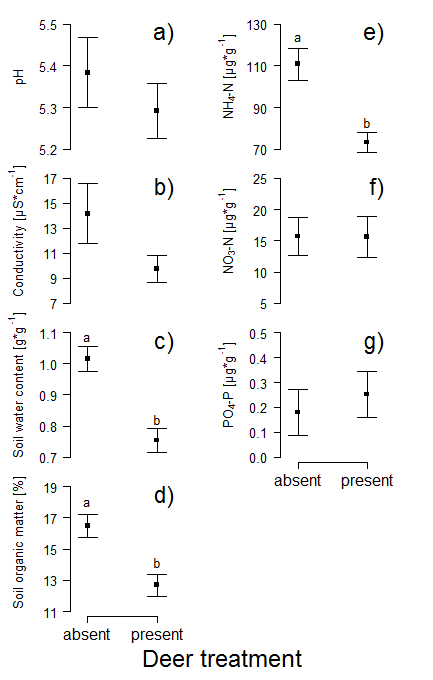


Abiotic soil conditions (mean ± SE) measured in plots in the deer present and deer absent treatments (n = 12 plots for each treatment). Lower case letters indicate differences between deer treatments (p < 0.05; Tukey contrast).

**Long-term deer exclosure alters soil properties, plant traits, understory plant community and insect herbivory, but not the functional relationships among them**

Jörg G. Stephan, Fereshteh Pourazari, Kristina Tattersdill, Takuya Kobayashi, Keita Nishizawa and Jonathan R. De Long

**Online Resource 7: Biotic properties within and around the plots**


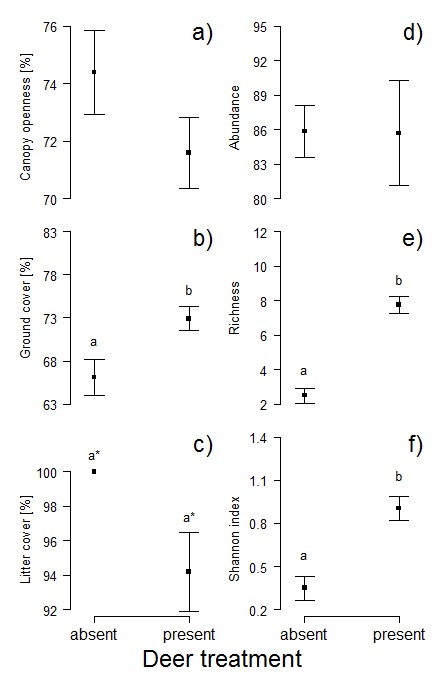


Biotic properties (mean ± SE) within and around the plots (a-c) and understory plant community measures (d-f) within the plots in the deer present and absent treatments (n=12 plots for each treatment). Different letters indicate significant differences between deer treatments (p < 0.05; Tukey contrast, except c that was compared with the Fishers exact test; star indicates strong tendency in panel c (p = 0.06)).

**Long-term deer exclosure alters soil properties, plant traits, understory plant community and insect herbivory, but not the functional relationships among them**

Jörg G. Stephan, Fereshteh Pourazari, Kristina Tattersdill, Takuya Kobayashi, Keita Nishizawa and Jonathan R. De Long

**Online Resource 8: Understory plant species percentage cover**

Understory plant species percentage cover (mean ± SE) in plots in the deer absent and deer present treatments (n = 12 for each treatment). Means are given only for plant species with 5% or greater cover in at least one plot.

| Understory plant species | Deer present | Deer absent |
| --- | --- | --- |
| *Aster ageratoides* | 19.1 ± 6.1 | 0.0 ± 0.0 |
| *Carex alopecuroides* var *chlorostachys* | 7.5 ± 2.0 | 0.0 ± 0.0 |
| *Sasa palmata* | 53.8 ± 5.5 | 74.2 ± 2.6 |
| *Thelypteris nipponica* | 0.4 ± 0.2 | 10.6 ± 3.1 |

**Long-term deer exclosure alters soil properties, plant traits, understory plant community and insect herbivory, but not the functional relationships among them**

Jörg G. Stephan, Fereshteh Pourazari, Kristina Tattersdill, Takuya Kobayashi, Keita Nishizawa and Jonathan R. De Long

**Online Resource 9: Tree diversity in the study area**

Cumulative tree abundance and species diversity within five meters of each deer absent or deer present treatment plots (n = 12 plots for each treatment).

| Tree species | Deer present | Deer absent |
| --- | --- | --- |
| *Betula playphylla* var *japonica* | 12 | 2 |
| *Euonymous sieboldianus* var *sanguineus* | 2 | 0 |
| *Malus toringo* | 0 | 12 |
| *Quercus crispula* | 72 | 74 |
| *Ulmus davidiana* var *japonica* | 1 | 6 |
| Total abundance | 87 | 94 |
| Shannon Index | 0.59 | 0.71 |
| Evenness | 0.41 | 0.51 |

**Long-term deer exclosure alters soil properties, plant traits, understory plant community and insect herbivory, but not the functional relationships among them**

Jörg G. Stephan, Fereshteh Pourazari, Kristina Tattersdill, Takuya Kobayashi, Keita Nishizawa and Jonathan R. De Long

**Online Resource 10: Diameter of trees in the study area**


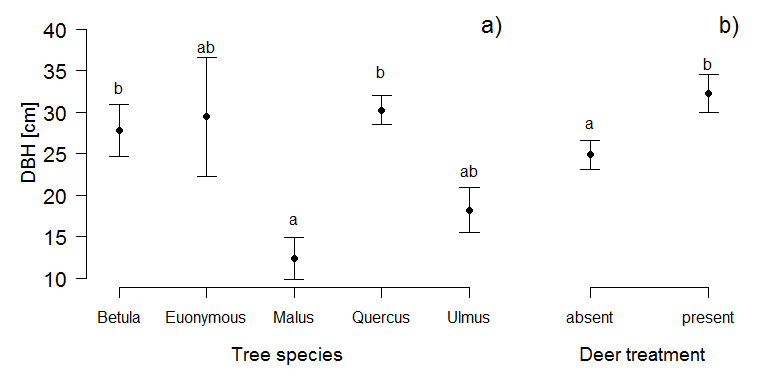


Tree diameter at breast height (DBH; if a tree had more than one stem, the diameter of all stems was summed) was significantly different between species (a; LM after log transformation and removal of non-significant interaction: F-value = 7.1, p = 0.001) and deer absent and present treatments (b; F-value = 1.5, p = 0.045). Letters indicate significant differences between tree species (see Online Resource 9 for full name) and deer present and absent treatments (p < 0.050; Tukey contrast).

**Long-term deer exclosure alters soil properties, plant traits, understory plant community and insect herbivory, but not the functional relationships among them**

Jörg G. Stephan, Fereshteh Pourazari, Kristina Tattersdill, Takuya Kobayashi, Keita Nishizawa and Jonathan R. De Long

**Online Resource 11: Plant and leaf traits of *Sasa palmata***


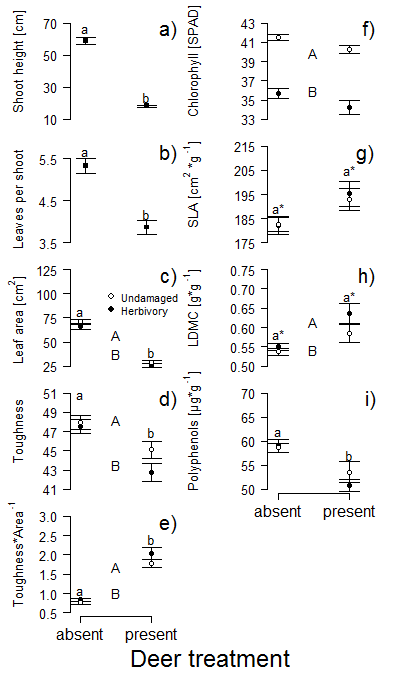


Plant and leaf traits (mean ± SE) measured on five *Sasa palmata* plants per plot in relation to deer present and absent treatments (n = 12 plots for each treatment) and insect herbivory (chewing, mining, or both; see text). Because leaf toughness increased with leaf size (Online Resource 3), the measured relative values were divided by the leaf area of the respective leaf. Different upper case letters indicate significant differences between herbivory damaged and undamaged leaves and different lowercase letters indicate significant differences between deer treatments (p < 0.05; Tukey contrast; star indicates strong tendency in panels g (p = 0.091) and h (p = 0.073)). LDMC = leaf dry matter content; SLA = specific leaf area; SPAD = portable chlorophyll meter measurements.

**Long-term deer exclosure alters soil properties, plant traits, understory plant community and insect herbivory, but not the functional relationships among them**

Jörg G. Stephan, Fereshteh Pourazari, Kristina Tattersdill, Takuya Kobayashi, Keita Nishizawa and Jonathan R. De Long

**Online Resource 12: Path coefficients of partial least square path models for deer absence and presence**


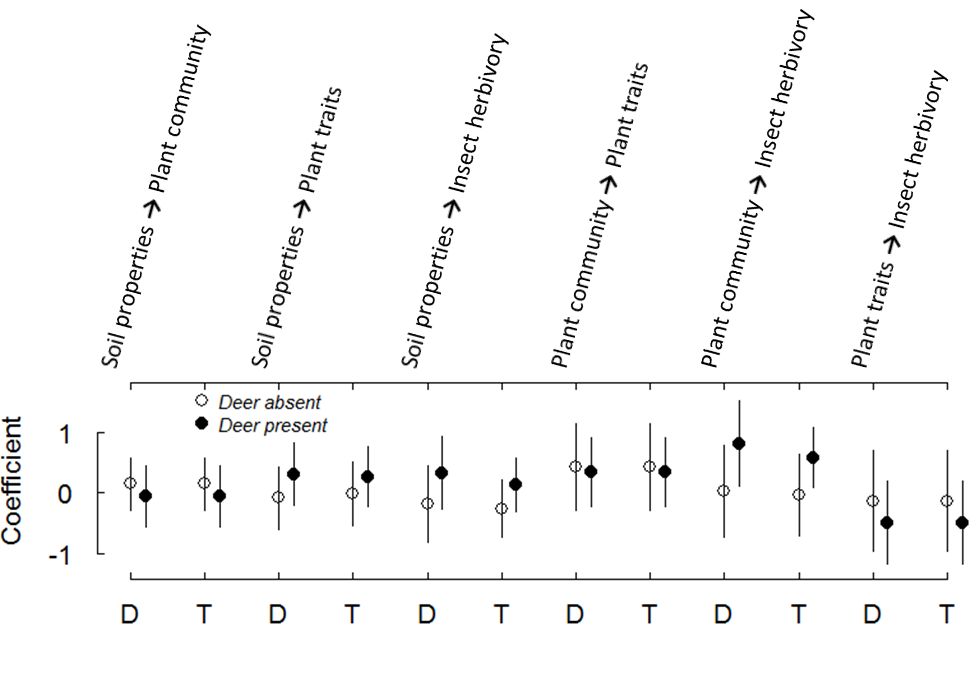


Bootstrapped coefficients (±SD) of direct (D) and total (T) effects for all six paths and deer presence or deer absence treatment. Comparing the respective coefficients for both treatments revealed no significant differences (t-test using mean, SD, and sample size (n = 12 for each treatment)) between path coefficients.
